# 5-Dichloro­acetyl-4-methyl-2,3,4,5-tetra­hydro-1*H*-1,5-benzodiazepin-2-one hemihydrate

**DOI:** 10.1107/S1600536809036940

**Published:** 2009-09-26

**Authors:** K. Ravichandran, P. Sakthivel, S. Ponnuswamy, M. Shalini, M. N. Ponnuswamy

**Affiliations:** a Centre of Advanced Study in Crystallography and Biophysics, University of Madras, Guindy Campus, Chennai 600 025, India; b Department of Chemistry, Government Arts College (Autonomous), Coimbatore 641 018, India

## Abstract

There are two crystallographically independent organic mol­ecules in the asymmetric unit of the title compound, C_12_H_12_Cl_2_N_2_O_2_·0.5H_2_O. The benzodiazepine ring adopts a distorted boat conformation in both molecules. The crystal packing is controlled by N—H⋯O, C—H⋯O and O—H⋯O intra- and inter­molecular hydrogen bonds. A graph-set motif of *R* ^3^_3_(14) dimer formation by a combination of N—H⋯O, O—H⋯O and C—H⋯O hydrogen bonds stabilizes the mol­ecules and extends along *a* axis.

## Related literature

For the anti­convulsant activity of benzodiazepine, see: MacDonald (2002 ▶). For their hypnotic effect, see: Gringauz (1999 ▶). For their use in the treatment of gastrointestinal and central nervous system disorders, see: Rahbaek *et al.* (1999 ▶). For other therapeutic applications, see: Albright *et al.* (1998 ▶); Lee *et al.* (1999 ▶). For hydrogen-bond motifs, see: Bernstein *et al.* (1995 ▶). For puckering and asymmetry parameters, see: Cremer & Pople (1975 ▶); Nardelli (1983 ▶). For details of the preparation of the title compound, see: Venkatraj *et al.* (2008 ▶).
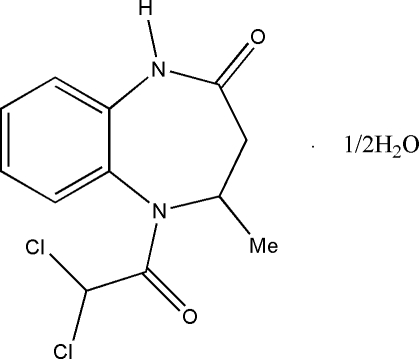


## Experimental

### 

#### Crystal data

C_12_H_12_Cl_2_N_2_O_2_·0.5H_2_O
*M* *_r_* = 592.29
Monoclinic, 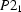

*a* = 8.5470 (3) Å
*b* = 18.0837 (6) Å
*c* = 8.8697 (3) Å
β = 95.405 (2)°
*V* = 1364.82 (8) Å^3^
*Z* = 2
Mo *K*α radiation
μ = 0.48 mm^−1^
*T* = 293 K
0.26 × 0.24 × 0.22 mm

#### Data collection

Bruker Kappa APEXII area-detector diffractometer
Absorption correction: multi-scan (*SADABS*; Sheldrick, 2001 ▶) *T*_min_ = 0.884, *T*_max_ = 0.901
14191 measured reflections
5599 independent reflections
4873 reflections with *I* > 2σ(*I*)
*R*_int_ = 0.022

#### Refinement

*R*[*F* ^2^ > 2σ(*F* ^2^)] = 0.045
*wR*(*F* ^2^) = 0.110
*S* = 1.04
5599 reflections
352 parameters
1 restraint
H atoms treated by a mixture of independent and constrained refinement
Δρ_max_ = 0.46 e Å^−3^
Δρ_min_ = −0.62 e Å^−3^
Absolute structure: Flack (1983 ▶), 2698 Friedel pairs
Flack parameter: 0.06 (6)

### 

Data collection: *APEX2* (Bruker, 2004 ▶); cell refinement: *SAINT* (Bruker, 2004 ▶); data reduction: *SAINT*; program(s) used to solve structure: *SHELXS97* (Sheldrick, 2008 ▶); program(s) used to refine structure: *SHELXL97* (Sheldrick, 2008 ▶); molecular graphics: *ORTEP-3* (Farrugia, 1997 ▶); software used to prepare material for publication: *SHELXL97* and *PLATON* (Spek, 2009 ▶).

## Supplementary Material

Crystal structure: contains datablocks global, I. DOI: 10.1107/S1600536809036940/bt5048sup1.cif

Structure factors: contains datablocks I. DOI: 10.1107/S1600536809036940/bt5048Isup2.hkl

Additional supplementary materials:  crystallographic information; 3D view; checkCIF report

## Figures and Tables

**Table 1:** Hydrogen-bond geometry (Å, °)

*D*—H⋯*A*	*D*—H	H⋯*A*	*D*⋯*A*	*D*—H⋯*A*
N1*A*—H1*A*⋯O3	0.87 (4)	2.08 (4)	2.927 (4)	164 (3)
O3—H2*W*⋯O1*B*^i^	0.80 (4)	2.02 (4)	2.815 (4)	173 (4)
C8*A*—H8*A*⋯O2*A*^ii^	0.93	2.51	3.268 (4)	139
C10*B*—H10*B*⋯O2*B*^iii^	0.93	2.39	3.179 (4)	143

Symmetry codes: (i) 
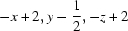
; (ii) 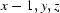; (iii) 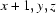.

**Fig. 1.**
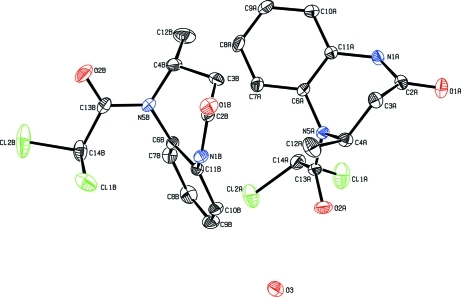
Perspective view of the molecule showing the thermal ellipsoids are drawn at 30% probability level.

**Fig. 2.**
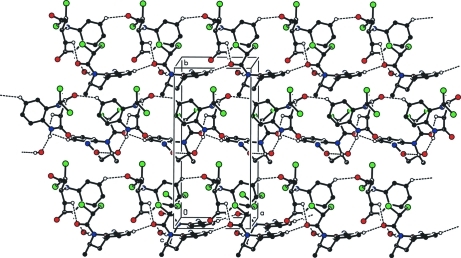
The crystal packing of the molecules viewed down c–axis. H atoms not involved in hydrogen bonding have been omitted for clarity.

## supplementary crystallographic information

### Comment

The anticonvulsant activity of benzodiazepines has been utilized clinically in patients to treat specific seizure types or conditions, *i.e.*, akinetic, myoclonic, absence variant seizures as well as to help terminate status epilepticusor serial seizures (MacDonald, 2002). Benzodiazepines are used for the purpose of hypnotic effects, owing to their less toxic and less severe withdrawal effects when compared with barbiturates (Gringauz, 1999). Benzodiazepines from *aspergillus* include *asperlicin*, which is used for treatment of gastrointestinal and central nervous system (*CNS*) disorders (Rahbaek *et al.*,1999). The other therapeutic applications (Lee *et al.*, 1999) of benzodiazepines include vasopressin antagonists (Albright *et al.*, 1998). In view of these importance and to ascertain the molecular conformation, crystallographic study of the title compound has been carried out.

The *ORTEP* diagram of the title compound is shown in Fig.1. There are two crystallographically independent molecules in the asymmetric unit. The benzodiazepine rings in the two molecules adopt a distorted boat conformation. The puckering parameters (Cremer & Pople, 1975) and the asymmetry parameters (Nardelli, 1983) for the ring in molecule A are: q_2_ = 0.959 (3) Å, q_3_ = 0.150 (3) Å, φ_2_ = 136.1 (2)°, φ_3_ = 359.8 (1)° and Δ2(C4A)= 8.1 (3)°; for the ring in molecule B are: q_2_ = 0.962 (3) Å, q_3_ = 0.168 (3) Å, φ_2_ = 141.4 (2)°, φ_3_ = 5.3 (1)° and Δ2(C4B)= 3.4 (3)°. The sum of the bond angles at N1A(359.0°), N1B(359.2), N5A(358.8) and N5B(359.9°) of the benzodiazepine rings in both the molecules are in accordance with *sp*^2^ hybridization.

The crystal packing is controlled by N—H···O, C—H···O and O—H···O types of intermolecular interactions in addition to van der Waals forces. The water molecule connects the molecules A and B through N1A—H1A···O3 and O3—H2W···O1B hydrogen bonds. Thus the combination of N1A—H1A···O3, O3—H2W···O1B and C3A—H3A···O2B hydrogen bonds form a graph set motif of *R*^3^_3_(14) dimer (Bernstein *et al.*, 1995) which stabilize the molecules. Atom C8A at (*x*, *y*, *z*) donates a proton to O2A (*x* - 1, *y*, *z*), which forms a C7 one dimensional chain running along a–axis. The intermolecular hydrogen bond C10B—H10B···O2B also connects the molecule into an another C7 chain running along b–axis (Fig. 2).

### Experimental

To a solution of tetrahydro-4-methyl-1,5-benzodiazepin-2-one (0.88 g) in anhydrous benzene (50 ml) was added triethylamine (2.8 ml) and dichloroacetyl chloride (1.90 ml). The contents were allowed to reflux on a water bath for 6hrs. The reaction mixture was washed with sodium bicarbonate solution (10%), water and dried. Evaporation of the solvent results a crude mass and further crystallization from ethanol gives colorless crystals (Venkatraj *et al.*, 2008).

### Refinement

The Nitrogen and Oxygen H atoms were refined and the other H atoms positioned geometrically (C—H=0.93–0.98 Å) and allowed to ride on their parent atoms, with 1.5*U*_eq_(C) for methyl H and 1.2 *U*_eq_(C) for other H atoms.

### Crystal data

**Table:**

C_12_H_12_Cl_2_N_2_O_2_·0.5H_2_O	*F*(000) = 612
*M**_r_* = 592.29	*D*_x_ = 1.441 Mg m^−^^3^
Monoclinic, *P*2_1_	Mo *K*α radiation, λ = 0.71073 Å
Hall symbol: P 2yb	Cell parameters from 4629 reflections
*a* = 8.5470 (3) Å	θ = 2.3–26.5°
*b* = 18.0837 (6) Å	µ = 0.48 mm^−^^1^
*c* = 8.8697 (3) Å	*T* = 293 K
β = 95.405 (2)°	Block, colourless
*V* = 1364.82 (8) Å^3^	0.26 × 0.24 × 0.22 mm
*Z* = 2	

### Data collection

**Table:**

Bruker Kappa APEXII area-detector diffractometer	5599 independent reflections
Radiation source: fine-focus sealed tube	4873 reflections with *I* > 2σ(*I*)
graphite	*R*_int_ = 0.022
ω and φ scans	θ_max_ = 26.5°, θ_min_ = 2.3°
Absorption correction: multi-scan (*SADABS*; Sheldrick, 2001)	*h* = −10→10
*T*_min_ = 0.884, *T*_max_ = 0.901	*k* = −22→22
14191 measured reflections	*l* = −11→10

### Refinement

**Table:**

Refinement on *F*^2^	Secondary atom site location: difference Fourier map
Least-squares matrix: full	Hydrogen site location: inferred from neighbouring sites
*R*[*F*^2^ > 2σ(*F*^2^)] = 0.045	H atoms treated by a mixture of independent and constrained refinement
*wR*(*F*^2^) = 0.110	*w* = 1/[σ^2^(*F*_o_^2^) + (0.0438*P*)^2^ + 0.7028*P*] where *P* = (*F*_o_^2^ + 2*F*_c_^2^)/3
*S* = 1.04	(Δ/σ)_max_ = 0.006
5599 reflections	Δρ_max_ = 0.46 e Å^−^^3^
352 parameters	Δρ_min_ = −0.62 e Å^−^^3^
1 restraint	Absolute structure: Flack (1983),2698 Friedel pairs
Primary atom site location: structure-invariant direct methods	Flack parameter: 0.06 (6)

### Special details

**Table:**

Geometry. All e.s.d.'s (except the e.s.d. in the dihedral angle between two l.s. planes) are estimated using the full covariance matrix. The cell e.s.d.'s are taken into account individually in the estimation of e.s.d.'s in distances, angles and torsion angles; correlations between e.s.d.'s in cell parameters are only used when they are defined by crystal symmetry. An approximate (isotropic) treatment of cell e.s.d.'s is used for estimating e.s.d.'s involving l.s. planes.
Refinement. Refinement of *F*^2^ against ALL reflections. The weighted *R*-factor *wR* and goodness of fit *S* are based on *F*^2^, conventional *R*-factors *R* are based on *F*, with *F* set to zero for negative *F*^2^. The threshold expression of *F*^2^ > σ(*F*^2^) is used only for calculating *R*-factors(gt) *etc*. and is not relevant to the choice of reflections for refinement. *R*-factors based on *F*^2^ are statistically about twice as large as those based on *F*, and *R*- factors based on ALL data will be even larger.

### Fractional atomic coordinates and isotropic or equivalent isotropic displacement parameters (Å^2^)

**Table:**

	*x*	*y*	*z*	*U*_iso_*/*U*_eq_	
Cl1A	1.62028 (16)	−0.29670 (7)	1.77856 (13)	0.0941 (4)	
Cl1B	1.04826 (15)	0.13662 (6)	0.96558 (15)	0.0906 (4)	
Cl2A	1.48485 (16)	−0.15064 (6)	1.74118 (13)	0.0858 (3)	
Cl2B	0.8172 (2)	0.15716 (9)	1.17529 (14)	0.1300 (7)	
O1A	1.5519 (3)	−0.46993 (14)	1.2925 (3)	0.0594 (6)	
O1B	1.0719 (3)	−0.07368 (15)	0.6539 (2)	0.0573 (6)	
O2A	1.6504 (3)	−0.20942 (14)	1.4809 (3)	0.0635 (7)	
O2B	0.7429 (3)	0.02505 (17)	1.0049 (4)	0.0852 (9)	
O3	1.2224 (4)	−0.56267 (15)	1.5161 (4)	0.0677 (7)	
H1W	1.279 (6)	−0.601 (3)	1.502 (6)	0.108 (19)*	
H2W	1.140 (5)	−0.570 (2)	1.467 (4)	0.059 (11)*	
N1A	1.3405 (3)	−0.42583 (13)	1.3919 (3)	0.0403 (5)	
H1A	1.325 (4)	−0.469 (2)	1.432 (4)	0.057 (10)*	
N1B	1.2097 (3)	−0.03383 (14)	0.8658 (3)	0.0399 (5)	
H1B	1.261 (3)	−0.0095 (17)	0.811 (3)	0.031 (7)*	
C2A	1.4518 (3)	−0.42199 (16)	1.2939 (3)	0.0420 (6)	
C2B	1.1026 (3)	−0.07798 (16)	0.7907 (3)	0.0410 (6)	
C3A	1.4398 (4)	−0.35821 (17)	1.1848 (3)	0.0462 (7)	
H3A	1.3314	−0.3539	1.1423	0.055*	
H3B	1.5028	−0.3694	1.1023	0.055*	
C3B	1.0275 (4)	−0.13513 (16)	0.8847 (3)	0.0465 (7)	
H3C	1.1089	−0.1594	0.9505	0.056*	
H3D	0.9773	−0.1723	0.8178	0.056*	
C4A	1.4920 (3)	−0.28414 (16)	1.2521 (3)	0.0441 (7)	
H4A	1.6071	−0.2832	1.2637	0.053*	
C4B	0.9057 (4)	−0.10270 (17)	0.9813 (4)	0.0490 (7)	
H4B	0.8130	−0.0886	0.9140	0.059*	
N5A	1.4357 (2)	−0.27668 (12)	1.4044 (2)	0.0368 (5)	
N5B	0.9699 (3)	−0.03578 (14)	1.0580 (2)	0.0403 (5)	
C6A	1.2763 (3)	−0.29648 (16)	1.4220 (3)	0.0355 (5)	
C6B	1.1307 (3)	−0.03545 (15)	1.1206 (3)	0.0363 (6)	
C7A	1.1664 (3)	−0.24204 (17)	1.4443 (3)	0.0457 (7)	
H7A	1.1961	−0.1926	1.4462	0.055*	
C7B	1.1690 (4)	−0.03548 (18)	1.2758 (3)	0.0485 (7)	
H7B	1.0906	−0.0415	1.3407	0.058*	
C8A	1.0148 (3)	−0.2607 (2)	1.4635 (4)	0.0548 (8)	
H8A	0.9424	−0.2242	1.4820	0.066*	
C8B	1.3225 (4)	−0.0267 (2)	1.3344 (4)	0.0587 (9)	
H8B	1.3482	−0.0259	1.4386	0.070*	
C9A	0.9695 (3)	−0.3334 (2)	1.4554 (4)	0.0530 (8)	
H9A	0.8658	−0.3458	1.4675	0.064*	
C9B	1.4383 (4)	−0.01891 (19)	1.2369 (4)	0.0571 (9)	
H9B	1.5418	−0.0109	1.2758	0.069*	
C10A	1.0749 (3)	−0.38800 (17)	1.4298 (4)	0.0467 (7)	
H10A	1.0422	−0.4370	1.4237	0.056*	
C10B	1.4017 (3)	−0.02294 (17)	1.0826 (4)	0.0461 (7)	
H10B	1.4812	−0.0194	1.0182	0.055*	
C11A	1.2304 (3)	−0.37036 (15)	1.4130 (3)	0.0353 (5)	
C11B	1.2482 (3)	−0.03216 (15)	1.0231 (3)	0.0363 (5)	
C12A	1.4358 (6)	−0.2213 (2)	1.1517 (4)	0.0707 (11)	
H12A	1.3230	−0.2210	1.1396	0.106*	
H12B	1.4751	−0.2271	1.0544	0.106*	
H12C	1.4733	−0.1755	1.1964	0.106*	
C12B	0.8546 (5)	−0.1577 (3)	1.0958 (5)	0.0806 (12)	
H12D	0.9429	−0.1705	1.1660	0.121*	
H12E	0.8147	−0.2015	1.0443	0.121*	
H12F	0.7738	−0.1361	1.1498	0.121*	
C13A	1.5282 (3)	−0.23943 (16)	1.5082 (3)	0.0425 (6)	
C13B	0.8785 (3)	0.02480 (19)	1.0567 (3)	0.0483 (7)	
C14A	1.4827 (4)	−0.2412 (2)	1.6705 (3)	0.0516 (7)	
H14A	1.3773	−0.2624	1.6720	0.062*	
C14B	0.9587 (5)	0.0969 (2)	1.1152 (4)	0.0651 (10)	
H14B	1.0379	0.0860	1.1995	0.078*	

### Atomic displacement parameters (Å^2^)

**Table:**

	*U*^11^	*U*^22^	*U*^33^	*U*^12^	*U*^13^	*U*^23^
Cl1A	0.1134 (9)	0.0939 (8)	0.0740 (7)	0.0444 (7)	0.0047 (6)	0.0100 (6)
Cl1B	0.1093 (9)	0.0570 (6)	0.1045 (8)	−0.0225 (6)	0.0047 (7)	0.0035 (6)
Cl2A	0.1158 (9)	0.0691 (6)	0.0740 (6)	0.0202 (6)	0.0169 (6)	−0.0282 (5)
Cl2B	0.1609 (13)	0.1493 (13)	0.0748 (7)	0.1052 (12)	−0.0141 (7)	−0.0412 (8)
O1A	0.0640 (14)	0.0558 (13)	0.0609 (13)	0.0228 (12)	0.0193 (11)	0.0018 (11)
O1B	0.0562 (13)	0.0768 (16)	0.0377 (11)	−0.0032 (12)	−0.0020 (9)	−0.0029 (11)
O2A	0.0445 (12)	0.0707 (16)	0.0778 (16)	−0.0228 (11)	0.0190 (11)	−0.0222 (13)
O2B	0.0332 (12)	0.087 (2)	0.133 (3)	0.0137 (13)	−0.0012 (14)	0.0200 (19)
O3	0.0578 (16)	0.0485 (14)	0.093 (2)	0.0005 (13)	−0.0113 (15)	0.0087 (13)
N1A	0.0465 (13)	0.0319 (13)	0.0435 (13)	0.0045 (10)	0.0100 (10)	0.0037 (10)
N1B	0.0367 (12)	0.0480 (13)	0.0360 (12)	−0.0030 (11)	0.0087 (10)	0.0039 (11)
C2A	0.0474 (16)	0.0378 (15)	0.0408 (14)	0.0031 (13)	0.0035 (12)	−0.0043 (12)
C2B	0.0388 (14)	0.0439 (16)	0.0400 (15)	0.0031 (12)	0.0026 (11)	−0.0056 (12)
C3A	0.0575 (18)	0.0471 (16)	0.0348 (13)	0.0020 (14)	0.0083 (12)	−0.0023 (12)
C3B	0.0504 (17)	0.0371 (15)	0.0511 (16)	−0.0102 (13)	−0.0010 (13)	−0.0056 (12)
C4A	0.0461 (16)	0.0472 (17)	0.0404 (14)	−0.0062 (13)	0.0108 (12)	−0.0014 (13)
C4B	0.0382 (15)	0.0495 (18)	0.0588 (19)	−0.0087 (13)	0.0016 (13)	0.0007 (14)
N5A	0.0328 (11)	0.0392 (12)	0.0394 (11)	−0.0022 (9)	0.0082 (9)	−0.0032 (9)
N5B	0.0282 (11)	0.0521 (14)	0.0409 (12)	0.0018 (10)	0.0047 (9)	0.0010 (11)
C6A	0.0277 (12)	0.0417 (14)	0.0367 (13)	0.0022 (11)	0.0010 (10)	0.0018 (11)
C6B	0.0334 (13)	0.0375 (13)	0.0372 (13)	0.0031 (11)	−0.0004 (10)	0.0018 (11)
C7A	0.0420 (15)	0.0402 (16)	0.0552 (17)	0.0070 (12)	0.0057 (13)	0.0037 (13)
C7B	0.0540 (17)	0.0523 (17)	0.0390 (14)	0.0062 (14)	0.0031 (12)	0.0047 (13)
C8A	0.0372 (16)	0.057 (2)	0.071 (2)	0.0182 (14)	0.0091 (14)	0.0025 (16)
C8B	0.071 (2)	0.060 (2)	0.0419 (16)	0.0053 (18)	−0.0129 (15)	−0.0005 (15)
C9A	0.0292 (15)	0.065 (2)	0.0648 (19)	−0.0016 (14)	0.0056 (13)	0.0010 (17)
C9B	0.0434 (17)	0.057 (2)	0.066 (2)	−0.0052 (15)	−0.0182 (15)	0.0001 (16)
C10A	0.0376 (15)	0.0460 (17)	0.0563 (17)	−0.0072 (12)	0.0031 (13)	−0.0004 (13)
C10B	0.0328 (13)	0.0472 (17)	0.0568 (17)	−0.0013 (12)	−0.0033 (12)	0.0023 (14)
C11A	0.0356 (13)	0.0376 (14)	0.0326 (12)	−0.0005 (11)	0.0032 (10)	−0.0005 (11)
C11B	0.0342 (13)	0.0347 (13)	0.0393 (13)	0.0007 (11)	0.0009 (10)	0.0015 (11)
C12A	0.104 (3)	0.049 (2)	0.061 (2)	−0.0015 (19)	0.019 (2)	0.0172 (17)
C12B	0.083 (3)	0.075 (3)	0.087 (3)	−0.036 (2)	0.026 (2)	0.004 (2)
C13A	0.0333 (14)	0.0402 (15)	0.0551 (16)	−0.0060 (12)	0.0107 (12)	−0.0084 (13)
C13B	0.0353 (15)	0.0581 (19)	0.0525 (17)	0.0096 (14)	0.0086 (13)	0.0103 (15)
C14A	0.0420 (15)	0.064 (2)	0.0482 (16)	0.0011 (14)	0.0015 (12)	−0.0146 (15)
C14B	0.073 (2)	0.064 (2)	0.0539 (19)	0.0303 (18)	−0.0134 (17)	−0.0112 (16)

### Geometric parameters (Å, °)

**Table:**

Cl1A—C14A	1.759 (3)	N5B—C13B	1.345 (4)
Cl1B—C14B	1.748 (4)	N5B—C6B	1.432 (3)
Cl2A—C14A	1.754 (3)	C6A—C7A	1.388 (4)
Cl2B—C14B	1.748 (4)	C6A—C11A	1.393 (4)
O1A—C2A	1.219 (4)	C6B—C7B	1.385 (4)
O1B—C2B	1.220 (3)	C6B—C11B	1.387 (4)
O2A—C13A	1.221 (3)	C7A—C8A	1.365 (4)
O2B—C13B	1.206 (4)	C7A—H7A	0.9300
O3—H1W	0.86 (6)	C7B—C8B	1.374 (5)
O3—H2W	0.80 (4)	C7B—H7B	0.9300
N1A—C2A	1.349 (4)	C8A—C9A	1.370 (5)
N1A—C11A	1.400 (4)	C8A—H8A	0.9300
N1A—H1A	0.87 (4)	C8B—C9B	1.381 (5)
N1B—C2B	1.343 (4)	C8B—H8B	0.9300
N1B—C11B	1.403 (3)	C9A—C10A	1.370 (4)
N1B—H1B	0.81 (3)	C9A—H9A	0.9300
C2A—C3A	1.503 (4)	C9B—C10B	1.376 (5)
C2B—C3B	1.508 (4)	C9B—H9B	0.9300
C3A—C4A	1.516 (4)	C10A—C11A	1.389 (4)
C3A—H3A	0.9700	C10A—H10A	0.9300
C3A—H3B	0.9700	C10B—C11B	1.378 (4)
C3B—C4B	1.527 (5)	C10B—H10B	0.9300
C3B—H3C	0.9700	C12A—H12A	0.9600
C3B—H3D	0.9700	C12A—H12B	0.9600
C4A—N5A	1.482 (3)	C12A—H12C	0.9600
C4A—C12A	1.495 (5)	C12B—H12D	0.9600
C4A—H4A	0.9800	C12B—H12E	0.9600
C4B—N5B	1.469 (4)	C12B—H12F	0.9600
C4B—C12B	1.515 (5)	C13A—C14A	1.526 (4)
C4B—H4B	0.9800	C13B—C14B	1.540 (5)
N5A—C13A	1.338 (4)	C14A—H14A	0.9800
N5A—C6A	1.431 (3)	C14B—H14B	0.9800
			
H1W—O3—H2W	106 (5)	C7A—C8A—C9A	119.9 (3)
C2A—N1A—C11A	125.0 (2)	C7A—C8A—H8A	120.1
C2A—N1A—H1A	117 (2)	C9A—C8A—H8A	120.1
C11A—N1A—H1A	117 (2)	C7B—C8B—C9B	119.4 (3)
C2B—N1B—C11B	126.2 (3)	C7B—C8B—H8B	120.3
C2B—N1B—H1B	114 (2)	C9B—C8B—H8B	120.3
C11B—N1B—H1B	119 (2)	C10A—C9A—C8A	120.9 (3)
O1A—C2A—N1A	120.5 (3)	C10A—C9A—H9A	119.6
O1A—C2A—C3A	123.0 (3)	C8A—C9A—H9A	119.6
N1A—C2A—C3A	116.4 (3)	C10B—C9B—C8B	120.5 (3)
O1B—C2B—N1B	121.9 (3)	C10B—C9B—H9B	119.7
O1B—C2B—C3B	122.0 (3)	C8B—C9B—H9B	119.7
N1B—C2B—C3B	116.1 (2)	C9A—C10A—C11A	120.2 (3)
C2A—C3A—C4A	115.1 (2)	C9A—C10A—H10A	119.9
C2A—C3A—H3A	108.5	C11A—C10A—H10A	119.9
C4A—C3A—H3A	108.5	C9B—C10B—C11B	120.4 (3)
C2A—C3A—H3B	108.5	C9B—C10B—H10B	119.8
C4A—C3A—H3B	108.5	C11B—C10B—H10B	119.8
H3A—C3A—H3B	107.5	C10A—C11A—C6A	118.8 (2)
C2B—C3B—C4B	113.3 (2)	C10A—C11A—N1A	120.8 (3)
C2B—C3B—H3C	108.9	C6A—C11A—N1A	120.4 (2)
C4B—C3B—H3C	108.9	C10B—C11B—C6B	119.0 (3)
C2B—C3B—H3D	108.9	C10B—C11B—N1B	120.6 (3)
C4B—C3B—H3D	108.9	C6B—C11B—N1B	120.3 (2)
H3C—C3B—H3D	107.7	C4A—C12A—H12A	109.5
N5A—C4A—C12A	111.1 (3)	C4A—C12A—H12B	109.5
N5A—C4A—C3A	109.3 (2)	H12A—C12A—H12B	109.5
C12A—C4A—C3A	111.8 (3)	C4A—C12A—H12C	109.5
N5A—C4A—H4A	108.2	H12A—C12A—H12C	109.5
C12A—C4A—H4A	108.2	H12B—C12A—H12C	109.5
C3A—C4A—H4A	108.2	C4B—C12B—H12D	109.5
N5B—C4B—C12B	110.4 (3)	C4B—C12B—H12E	109.5
N5B—C4B—C3B	109.4 (2)	H12D—C12B—H12E	109.5
C12B—C4B—C3B	112.3 (3)	C4B—C12B—H12F	109.5
N5B—C4B—H4B	108.2	H12D—C12B—H12F	109.5
C12B—C4B—H4B	108.2	H12E—C12B—H12F	109.5
C3B—C4B—H4B	108.2	O2A—C13A—N5A	123.3 (3)
C13A—N5A—C6A	123.9 (2)	O2A—C13A—C14A	119.7 (3)
C13A—N5A—C4A	116.8 (2)	N5A—C13A—C14A	116.9 (2)
C6A—N5A—C4A	118.1 (2)	O2B—C13B—N5B	122.9 (3)
C13B—N5B—C6B	122.4 (3)	O2B—C13B—C14B	120.4 (3)
C13B—N5B—C4B	118.4 (2)	N5B—C13B—C14B	116.5 (3)
C6B—N5B—C4B	119.1 (2)	C13A—C14A—Cl2A	108.8 (2)
C7A—C6A—C11A	119.8 (2)	C13A—C14A—Cl1A	108.0 (2)
C7A—C6A—N5A	120.2 (3)	Cl2A—C14A—Cl1A	110.73 (17)
C11A—C6A—N5A	120.0 (2)	C13A—C14A—H14A	109.7
C7B—C6B—C11B	120.2 (2)	Cl2A—C14A—H14A	109.7
C7B—C6B—N5B	120.9 (3)	Cl1A—C14A—H14A	109.7
C11B—C6B—N5B	118.8 (2)	C13B—C14B—Cl2B	109.4 (3)
C8A—C7A—C6A	120.4 (3)	C13B—C14B—Cl1B	107.7 (2)
C8A—C7A—H7A	119.8	Cl2B—C14B—Cl1B	109.9 (2)
C6A—C7A—H7A	119.8	C13B—C14B—H14B	109.9
C8B—C7B—C6B	120.2 (3)	Cl2B—C14B—H14B	109.9
C8B—C7B—H7B	119.9	Cl1B—C14B—H14B	109.9
C6B—C7B—H7B	119.9		
			
C11A—N1A—C2A—O1A	173.3 (3)	C7B—C8B—C9B—C10B	−2.6 (5)
C11A—N1A—C2A—C3A	−9.3 (4)	C8A—C9A—C10A—C11A	0.6 (5)
C11B—N1B—C2B—O1B	177.0 (3)	C8B—C9B—C10B—C11B	2.3 (5)
C11B—N1B—C2B—C3B	−5.4 (4)	C9A—C10A—C11A—C6A	−0.2 (4)
O1A—C2A—C3A—C4A	−106.8 (3)	C9A—C10A—C11A—N1A	177.8 (3)
N1A—C2A—C3A—C4A	75.9 (3)	C7A—C6A—C11A—C10A	−1.4 (4)
O1B—C2B—C3B—C4B	−107.2 (3)	N5A—C6A—C11A—C10A	−179.7 (2)
N1B—C2B—C3B—C4B	75.2 (3)	C7A—C6A—C11A—N1A	−179.4 (2)
C2A—C3A—C4A—N5A	−40.6 (4)	N5A—C6A—C11A—N1A	2.2 (4)
C2A—C3A—C4A—C12A	−164.0 (3)	C2A—N1A—C11A—C10A	138.6 (3)
C2B—C3B—C4B—N5B	−46.2 (3)	C2A—N1A—C11A—C6A	−43.4 (4)
C2B—C3B—C4B—C12B	−169.2 (3)	C9B—C10B—C11B—C6B	1.6 (4)
C12A—C4A—N5A—C13A	−90.1 (3)	C9B—C10B—C11B—N1B	177.9 (3)
C3A—C4A—N5A—C13A	146.1 (3)	C7B—C6B—C11B—C10B	−5.2 (4)
C12A—C4A—N5A—C6A	78.1 (3)	N5B—C6B—C11B—C10B	172.3 (3)
C3A—C4A—N5A—C6A	−45.7 (3)	C7B—C6B—C11B—N1B	178.5 (3)
C12B—C4B—N5B—C13B	−102.0 (3)	N5B—C6B—C11B—N1B	−3.9 (4)
C3B—C4B—N5B—C13B	134.0 (3)	C2B—N1B—C11B—C10B	141.2 (3)
C12B—C4B—N5B—C6B	83.0 (3)	C2B—N1B—C11B—C6B	−42.6 (4)
C3B—C4B—N5B—C6B	−41.1 (3)	C6A—N5A—C13A—O2A	−164.1 (3)
C13A—N5A—C6A—C7A	58.9 (4)	C4A—N5A—C13A—O2A	3.3 (4)
C4A—N5A—C6A—C7A	−108.3 (3)	C6A—N5A—C13A—C14A	21.3 (4)
C13A—N5A—C6A—C11A	−122.8 (3)	C4A—N5A—C13A—C14A	−171.3 (3)
C4A—N5A—C6A—C11A	70.0 (3)	C6B—N5B—C13B—O2B	−179.7 (3)
C13B—N5B—C6B—C7B	75.4 (4)	C4B—N5B—C13B—O2B	5.4 (4)
C4B—N5B—C6B—C7B	−109.8 (3)	C6B—N5B—C13B—C14B	4.5 (4)
C13B—N5B—C6B—C11B	−102.1 (3)	C4B—N5B—C13B—C14B	−170.4 (3)
C4B—N5B—C6B—C11B	72.7 (3)	O2A—C13A—C14A—Cl2A	53.9 (3)
C11A—C6A—C7A—C8A	2.6 (4)	N5A—C13A—C14A—Cl2A	−131.2 (2)
N5A—C6A—C7A—C8A	−179.0 (3)	O2A—C13A—C14A—Cl1A	−66.3 (3)
C11B—C6B—C7B—C8B	4.9 (5)	N5A—C13A—C14A—Cl1A	108.5 (3)
N5B—C6B—C7B—C8B	−172.6 (3)	O2B—C13B—C14B—Cl2B	27.4 (4)
C6A—C7A—C8A—C9A	−2.3 (5)	N5B—C13B—C14B—Cl2B	−156.7 (2)
C6B—C7B—C8B—C9B	−1.0 (5)	O2B—C13B—C14B—Cl1B	−92.0 (3)
C7A—C8A—C9A—C10A	0.7 (5)	N5B—C13B—C14B—Cl1B	84.0 (3)

### Hydrogen-bond geometry (Å, °)

**Table:**

*D*—H···*A*	*D*—H	H···*A*	*D*···*A*	*D*—H···*A*
C4A—H4A···O2A	0.98	2.34	2.694 (4)	100
C4B—H4B···O2B	0.98	2.31	2.715 (4)	104
N1A—H1A···O3	0.87 (4)	2.08 (4)	2.927 (4)	164 (3)
O3—H2W···O1B^i^	0.80 (4)	2.02 (4)	2.815 (4)	173 (4)
C3A—H3A···O2B^i^	0.97	2.60	3.038 (4)	108
C8A—H8A···O2A^ii^	0.93	2.51	3.268 (4)	139
C10B—H10B···O2B^iii^	0.93	2.39	3.179 (4)	143

Symmetry codes:  (i) −*x*+2, *y*−1/2, −*z*+2; (ii) *x*−1, *y*, *z*; (iii) *x*+1, *y*, *z*.

## References

[bb1] Albright, J. D., Feich, M. F., Santos, E. G. D., Dusza, J. P., Sum, F.-W., Venkatesan, A. M., Coupet, J., Chan, P. S., Ru, X., Mazandarani, H. & Bailey, T. (1998). *J. Med. Chem.* **41**, 2442–2444. 10.1021/jm980179c 9651149

[bb2] Bernstein, J., Davis, R. E., Shimoni, L. & Chang, N. L. (1995). *Angew. Chem. Int. Ed. Engl.* **34**, 1555–1573.

[bb3] Bruker (2004). *APEX2* and *SAINT* Bruker AXS Inc. Madison, Wisconsin, USA.

[bb4] Cremer, D. & Pople, J. A. (1975). *J. Am. Chem. Soc.* **97**, 1354–1358.

[bb5] Farrugia, L. J. (1997). *J. Appl. Cryst.* **30**, 565.

[bb6] Flack, H. D. (1983). *Acta Cryst.* A**39**, 876–881.

[bb7] Gringauz, A. (1999). *Introduction to Medicinal Chemistry*, pp. 578–580. New York: Wiley-VCH.

[bb8] Lee, J., Gauthier, D. & Rivero, R. A. (1999). *J. Org. Chem*, **64**, 3060–3064. 10.1021/jo981620+ 11674402

[bb9] MacDonald, R. L. (2002). *Benzodiazepines Mechanisms of Action. *In* Antiepileptic Drugs, 5th* ed., edited by R. H. Levy, R. H. Mattson, B. S. Meldrum & E. Perucca, pp. 179–186. Philadelphia: Lippincott Williams and Wilkins.

[bb10] Nardelli, M. (1983). *Acta Cryst.* C**39**, 1141–1142.

[bb11] Rahbaek, L., Breinholt, J., Frisvad, J. C. & Christophersen, C. (1999). *J. Org. Chem.* **64**, 1689–1692. 10.1021/jo981536u 11674237

[bb12] Sheldrick, G. M. (2001). *SADABS* University of Göttingen, Germany.

[bb13] Sheldrick, G. M. (2008). *Acta Cryst.* A**64**, 112–122. 10.1107/S0108767307043930 18156677

[bb14] Spek, A. L. (2009). *Acta Cryst* D**65**, 148–155. 10.1107/S090744490804362X PMC2631630 19171970

[bb15] Venkatraj, M., Ponnuswamy, S. & Jeyaraman, R. (2008). *Indian J. Chem. Sect. B*, **47**, 129–135.

